# Risk of Atrial Fibrillation Recurrence After Modified Cox-Maze IV Surgery: Comparing Atrial Functional Mitral Regurgitation and Rheumatic Mitral Regurgitation

**DOI:** 10.31083/RCM49184

**Published:** 2026-09-07

**Authors:** Ke Xu, Jingcheng Wu, Yi Li, Lailong Sun, Ming Yan, Fuwei Zhang, Chaobing Liu, SongLin Zhang

**Affiliations:** ^1^Department of Thoracic and Cardiovascular Surgery, First Clinical Medical College of China Three Gorges University & Yichang Central People’s Hospital, 443002 Yichang, Hubei, China

**Keywords:** atrial fibrillation, modified Cox-Maze IV procedure, mitral valve insufficiency, atrial functional mitral regurgitation, rheumatic mitral regurgitation, atrial fibrillation recurrence

## Abstract

**Background:**

The comparative efficacy of the modified Cox-Maze IV procedure (CMP IV) in treating atrial fibrillation (AF) in patients with atrial functional mitral regurgitation (AFMR) and those with rheumatic mitral regurgitation (RMR) remains inadequately characterized. Therefore, this study aimed to evaluate and compare postoperative AF recurrence rates and clinical outcomes between these two etiologically distinct groups.

**Methods:**

A retrospective analysis was conducted on 221 patients (AFMR group: n = 53; RMR group: n = 168) with concomitant long-standing persistent AF who underwent mitral valve surgery combined with modified CMP IV or other concomitant procedures at our institution between January 2019 and September 2024. To address baseline imbalances, propensity score matching was performed at a 1:1 ratio, resulting in well-matched cohorts of 32 patients with AFMR and 32 with RMR patients for the final analysis. The primary endpoint was AF recurrence. Statistical comparisons utilized chi-square tests, *t*-tests, exact McNemar tests, and paired *t*-tests.

**Results:**

After PSM, 32 patients remained in each group with balanced baselines. The AFMR group showed a higher trend toward postoperative AF recurrence. Pre‑discharge AF recurrence rates did not differ significantly between groups (37.5% vs. 12.5%; *p* = 0.057), however, the risk of AF recurrence before discharge was significantly higher in the AFMR group (OR = 3.67, 95% CI: 1.02–13.14, *p* = 0.046). At the 1-year follow-up, a statistically significant difference in AF recurrence was observed between the two groups (31.2% vs 6.2%, *p* = 0.021), and the risk difference persisted (OR = 9.00, 95% CI: 1.14–71.04, *p* = 0.037). Kaplan–Meier analysis showed a significant difference in freedom from AF recurrence (log‑rank χ^2^ = 6.33; *p* = 0.012), supported by sensitivity analyses (log‑rank χ^2^ = 6.01;* p* = 0.014). Mortality did not differ at 30 days (3.1% vs. 0%;* p* = 1.000) or 1 year (3.1% vs. 3.1%; *p *= 1.000), with comparable overall survival (log‑rank *p* = 0.99).

**Conclusions:**

In patients undergoing mitral valve surgery with modified CMP IV, those with AFMR exhibit a significantly higher risk of postoperative AF recurrence compared with patients with RMR, despite similar early-term survival outcomes. Postoperative AF monitoring should be intensified in patients with AFMR to improve long-term outcomes.

## 1. Introduction

Atrial fibrillation (AF) represents one of the most prevalent clinical arrhythmias [1], often necessitating active intervention upon progression to persistent or longstanding forms [2]. The modified Cox-Maze IV procedure (CMP IV), introduced by the Cox team, is now widely adopted for such patients [3]. This technique involves targeted ablation of abnormal atrial conduction pathways, thereby confining electrical propagation to predefined routes, directing sinoatrial impulses appropriately to the atria and atrioventricular node, and eliminating macro–reentrant circuits. Its objective is to restore sinus rhythm and improve cardiac hemodynamics [4]. With progressive refinements in CMP IV, operative duration, complication rates, and postoperative AF recurrence have substantially declined, leading to its increasing integration with concomitant mitral valve surgery. Available evidence suggests that in patients with mitral valve disease (MVD) and concomitant AF, the addition of CMP IV adds minimal aortic cross-clamp time, does not increase overall surgical risk or rates of mortality, pacemaker implantation, stroke, or thromboembolism, and is associated with significantly lower postoperative AF recurrence compared with mitral valve surgery alone [3,5,6].

AF and MVD share a close pathophysiological relationship. Common etiologies of MVD include rheumatic heart disease, degenerative mitral regurgitation (DMR), infective endocarditis, and ischemic heart disease. In recent years, “atrial functional mitral regurgitation (AFMR)” has been recognized as a distinct entity, characterized by functional mitral regurgitation secondary to isolated AF in the absence of ventricular dysfunction. Early recognition of AFMR is clinically important, as it may influence the success of rhythm-control strategies such as catheter ablation. Advanced imaging modalities, including three-dimensional echocardiography, have demonstrated that marked mitral annular and left atrial dilation in AF patients frequently accompanies significant functional regurgitation. Proposed mechanisms involve atrial-mediated leaflet tethering, annular–leaflet area mismatch due to inadequate leaflet adaptation, and increased valvular stress from impaired annular contractility [7]. In contrast, traditional functional mitral regurgitation resulting from left ventricular systolic or diastolic dysfunction is classified as Carpentier type IIIb and termed “ventricular functional mitral regurgitation (VFMR)” [8].

Currently, there remains a paucity of comparative studies evaluating surgical outcomes in patients with AF and MVD of differing etiologies. More robust, regionally representative data are needed to inform evidence-based clinical management. This is particularly true for AFMR with concomitant AF, for which surgical evidence remains limited and no dedicated guidelines exist. This study was therefore designed to systematically assess sinus rhythm maintenance and clinical outcomes following modified CMP IV surgery in patients with AFMR, and to compare these results with those of a regionally prevalent cohort suffering from rheumatic mitral regurgitation (RMR) with AF.

## 2. Methods

### 2.1 Study Design and Population

This single-center retrospective cohort study compared surgical outcomes between patients with AFMR and those with RMR, both groups presenting with long-standing persistent atrial fibrillation. Baseline characteristics were summarized for all enrolled patients. To address potential confounding due to baseline imbalances, propensity score matching (PSM) was performed between the two etiological groups prior to comparative analysis of postoperative outcomes.

This study consecutively enrolled patients who underwent mitral valve surgery (repair or replacement) combined with a modified CMP IV at the Department of Cardiovascular Surgery, Yichang Central People's Hospital, from January 2019 to September 2024.

#### 2.1.1 Inclusion and Exclusion Criteria

Patients were included if they met the above surgical criteria. The following exclusion criteria were applied: (1) History of other significant cardiac comorbidities (e.g., severe coronary artery disease, cardiomyopathy, or congenital heart disease). (2) Concurrent cardiac procedures other than tricuspid valve repair, aortic valve replacement, or the Maze procedure (e.g., coronary artery bypass grafting). (3) Preoperative permanent pacemaker implantation. (4) Preoperative chronic renal insufficiency or hepatic dysfunction. (5) Inability to complete regular postoperative follow-up or premature withdrawal from the study.

#### 2.1.2 Definition of AFMR and RMR

Patients were categorized into either the AFMR group or the RMR group based on a comprehensive preoperative evaluation, which included clinical symptoms, physical signs, admission records, cardiac function classification, preoperative electrocardiography, preoperative echocardiography, and surgical records.

The diagnosis of AFMR was established according to the criteria proposed by Deferm et al. (2019) [9], requiring the fulfillment of all the following echocardiographic and hemodynamic criteria: (1) left atrial enlargement; (2) normal left ventricular morphology and size; (3) preserved left ventricular ejection fraction (LVEF >50%); and (4) mitral annular dilation.

RMR was diagnosed based on characteristic echocardiographic features [10]: (1) pathological mitral regurgitation, defined as a regurgitant jet seen in two views, with a jet length ≥2 cm, peak velocity ≥3 m/s, and a pan‑systolic Doppler signal in at least one view; (2) at least two morphological features of rheumatic mitral valve involvement, including anterior mitral valve leaflet thickening ≥3 mm, chordal thickening, restricted leaflet motion, or excessive leaflet tip motion during systole; (3) exclusion of other causes of mitral regurgitation, with the final diagnosis confirmed by an expert echocardiographer.

### 2.2 Surgical Technique

For mitral valve surgery, patients undergoing mitral valve replacement were included in this study, and all prosthetic valves used were complete rings manufactured by a unified supplier. According to preoperative echocardiographic findings, patients with moderate or greater regurgitation or stenosis of the tricuspid or aortic valves underwent intraoperative valve exploration. Valve repair was prioritized, and valve replacement was performed when repair was deemed unfeasible or unsatisfactory.

This study employed a modified CMP IV procedure. Ablation was primarily performed using bipolar radio frequency clamps, with six repeated applications along the pulmonary vein antrum to create transmural “X”-shaped lesions and four applications along other linear paths.

Right-sided ablation lines: In the right atrial lesion group, the procedure was performed under beating heart conditions after the establishment of cardiopulmonary bypass. The ablation lines were created as follows: (1) a right atriotomy was made on the free wall of the right atrium, at the junction of the superior and inferior vena cavae, approximately one-third of the distance toward the inferior vena cava; (2) a vertical ablation line was created from the superior vena cava ostium to the inferior vena cava ostium; (3) an ablation line was extended from the right atriotomy to the right atrial appendage; (4) an additional ablation line was created from the right atriotomy to the 2 o’ clock position of the tricuspid annulus (corresponding to the commissure between the anterior and posterior leaflets of the tricuspid valve). To protect the right coronary artery, the bipolar radiofrequency ablation clamp was carefully passed underneath the suspended right coronary artery before grasping the atrial tissue for ablation.

Left atrial procedures routinely included: right pulmonary vein antrum isolation before cardiopulmonary bypass; after aortic cross‑clamping, left pulmonary vein antrum isolation and roof/floor lines connecting the bilateral pulmonary veins to complete a box‑lesion set; left atrial appendage excision followed by ablation from its base toward the left pulmonary veins; and mitral isthmus ablation performed via a left atriotomy.

The mitral isthmus line was created using a modified approach: epicardial transection of the ligament of Marshall; endocardial ablation of the coronary sinus through the left atriotomy, ensuring overlap with the isthmus line; and a linear “cut‑and‑sew” incision from the endocardial aspect toward the mitral annulus in areas not accessible to the radiofrequency clamp.

All patients underwent concomitant mitral valve surgery (replacement or repair). Some patients received additional tricuspid or aortic valve procedures. All operations were performed by the same cardiac surgeon, following a standardized workflow with only minimal technical variations.

### 2.3 Data Collection

Patient baseline characteristics and examination results were extracted from the electronic medical records and outpatient registries of Yichang Central People’s Hospital. The following definitions were used:

Stroke: A documented history of cerebral infarction.

Coronary Heart Disease: Coronary artery stenosis exceeding 75%.

Hypertension [11]: (1) Grade Ⅰ hypertension, defined as systolic BP 140–159 mmHg and/or diastolic BP 90–99 mmHg; (2) Grade Ⅱ hypertension, defined as systolic BP 160–179 mmHg and/or diastolic BP 100–109 mmHg; and (3) Grade Ⅲ hypertension, defined as systolic BP ≥180 mmHg and/or diastolic BP ≥110 mmHg.

Arrhythmia: Patients with a clinical diagnosis of long-standing persistent atrial fibrillation.

Renal dysfunction: Serum creatinine (Scr) level >115 μmol/L. Postoperative acute kidney injury (AKI): A postoperative increase to ≥3 times the preoperative level.

Short-term Mortality (30-day mortality): Death occurring within 30 days postoperatively.

Early mortality (1-year mortality): Death occurring within one year after surgery.

Preoperative Transthoracic Echocardiography (TTE): The results of the final examination performed before surgery.

Pre-discharge electrocardiograms (ECG): The final electrocardiogram obtained prior to hospital discharge.

During follow-up, outpatient and inpatient ECG results (ECG or Holter monitoring) were recorded at 1, 3, 6, and 12 months postoperatively. The absence of atrial fibrillation recurrence was defined as two or more consecutive ECGs showing sinus rhythm. Overall survival was followed from the date of surgery until the date of last clinical contact or death, with a median follow‑up of 25–26 months. All atrial arrhythmias and permanent pacemaker rhythms were categorized as failure to restore sinus rhythm.

### 2.4 Statistical Analysis

Continuous variables are presented as mean ± standard deviation (SD) when normally distributed and as median (interquartile range, IQR) when non-normally distributed, with between-group comparisons performed using the independent-samples *t*‑test or Mann‑Whitney U test, with the test statistic reported. Categorical variables are expressed as frequency (percentage) and compared using the chi‑square test or Fisher’s exact test, as appropriate. All statistical tests were two‑tailed, with a *p*-value < 0.05 considered statistically significant.

Baseline characteristics were summarized for the entire cohort before matching. To minimize selection bias and balance baseline differences between the AFMR group and the rheumatic mitral regurgitation group (Table 1), PSM was performed. Propensity scores were estimated using a logistic regression model that included all clinically relevant baseline variables. Patients were matched 1:1 using the nearest‑neighbor method with a caliper width of 0.2. After matching, the standardized mean differences (SMD) of covariates between the two groups were calculated to assess the effect size of intergroup differences, with SMD <0.2 as the prespecified criterion for acceptable balance. The baseline comparability was assessed, and all subsequent outcome analyses were conducted on the matched population.

**Table 1.** **Baseline characteristics of the study population before propensity score matching**.

Characteristic			RMR group (N = 168)	AFMR group (N = 53)	*p-*values
Age, median (IQR), years			57.00 (11)	63.00 (10)	<0.001*
Sex (male/female)			34/134	30/23	<0.001*
BSA, median (IQR), m^2^			1.576 (0.19)	1.695 (0.28)	<0.001*
NYHA functional class, n (%)					0.204
	Class Ⅰ		4 (2.3)	1 (1.9)	
	Class Ⅱ		14 (8.3)	3 (5.7)	
	Class Ⅲ		128 (76.1)	36 (67.9)	
	Class Ⅳ		22 (13.0)	13 (24.5)	
Preoperative comorbidities, n (%)					
	Hypertension, n (%)				<0.001*
		Grade Ⅰ	9 (5.4)	3 (5.7)	
		Grade Ⅱ	2 (1.2)	4 (7.5)	
		Grade Ⅲ	8 (4.8)	10 (18.9)	
	Diabetes, n (%)		5 (3.0)	2 (3.8)	0.674
	Coronary artery disease, n (%)		14 (8.3)	8 (15.1)	0.152
	Renal dysfunction, n (%)		46 (27.4)	0 (0)	<0.001*
	Stroke, n (%)		4 (2.4)	0 (0)	0.575
	History of cardiac surgery, n (%)		2 (1.2)	1 (1.9)	0.563

Abbreviations: RMR, rheumatic mitral regurgitation; AFMR, atrial functional mitral regurgitation; IQR, interquartile range; BSA, body surface area; NYHA, New York Heart Association. Notes: Data are presented as median (IQR) or n (%). *p*-values were calculated using the Mann-Whitney U test for continuous variables and the Chi-square test or Fisher’s exact test for categorical variables, as appropriate. An asterisk (*) indicates a statistically significant difference (*p* < 0.05). Post‑hoc subgroup comparisons were not formally tested for multiplicity.

After matching, continuous variables with normal distribution were presented as mean ± SD and compared between groups using the paired *t*-test; continuous variables with non-normal distribution were presented as median (interquartile range) and compared using the Wilcoxon signed-rank test. Categorical variables were presented as counts and percentages (n, %) and compared using exact McNemar’s test or the marginal homogeneity test, as appropriate. To compare recurrence rates at a fixed time point (Before discharge and 1 year postoperatively) in this propensity score-matched cohort, the exact McNemar’s test was used, and the odds ratio (OR) with 95% confidence interval (CI) was calculated based on the conditional maximum likelihood estimation for matched pairs and presented in a forest plot. Survival curves were generated using the Kaplan-Meier method, and differences between survival curves were compared using the log-rank test. To assess the robustness of the results, we conducted sensitivity analyses that included a 3-month blanking period. The blanking period was defined as the first 3 months post-surgery; atrial fibrillation events occurring during this period were excluded from the recurrence endpoint. Three sensitivity analysis methods were employed: (1) excluding patients who experienced recurrence during the blanking period; (2) setting the start of follow-up at 3 months post-surgery (left-censored); (3) treating patients with recurrence during the blanking period as missing. Since the control group had no recurrence events after the blanking period, the Cox proportional hazards model could not estimate a reliable hazard ratio; therefore, the log-rank test was used to compare the differences in survival curves between the two groups. All tests were two-sided, and *p* < 0.05 was considered statistically significant.

To identify independent predictors of atrial fibrillation recurrence, univariate Cox regression was initially performed on the entire cohort. Variables with a *p*-value < 0.1 in univariate analysis were subsequently entered into a multivariate Cox proportional hazards model. Model assumptions were verified, and no major violations were detected.

All statistical analyses were performed using SPSS 27.0 (IBM Corp., Armonk, NY, USA) and R 4.4.3 (R Foundation for Statistical Computing, Vienna, Austria). Data organization, descriptive statistics, and matching procedures were implemented in R, while other analyses were conducted in SPSS.

## 3. Results

A total of 221 patients were included in the study, with 53 patients in the AFMR group and 168 patients in the RMR group (Fig. 1). Following 1:1 propensity score matching, the final cohort consisted of 32 patients in the AFMR group and 32 patients in the RMR group.

**Fig. 1.**
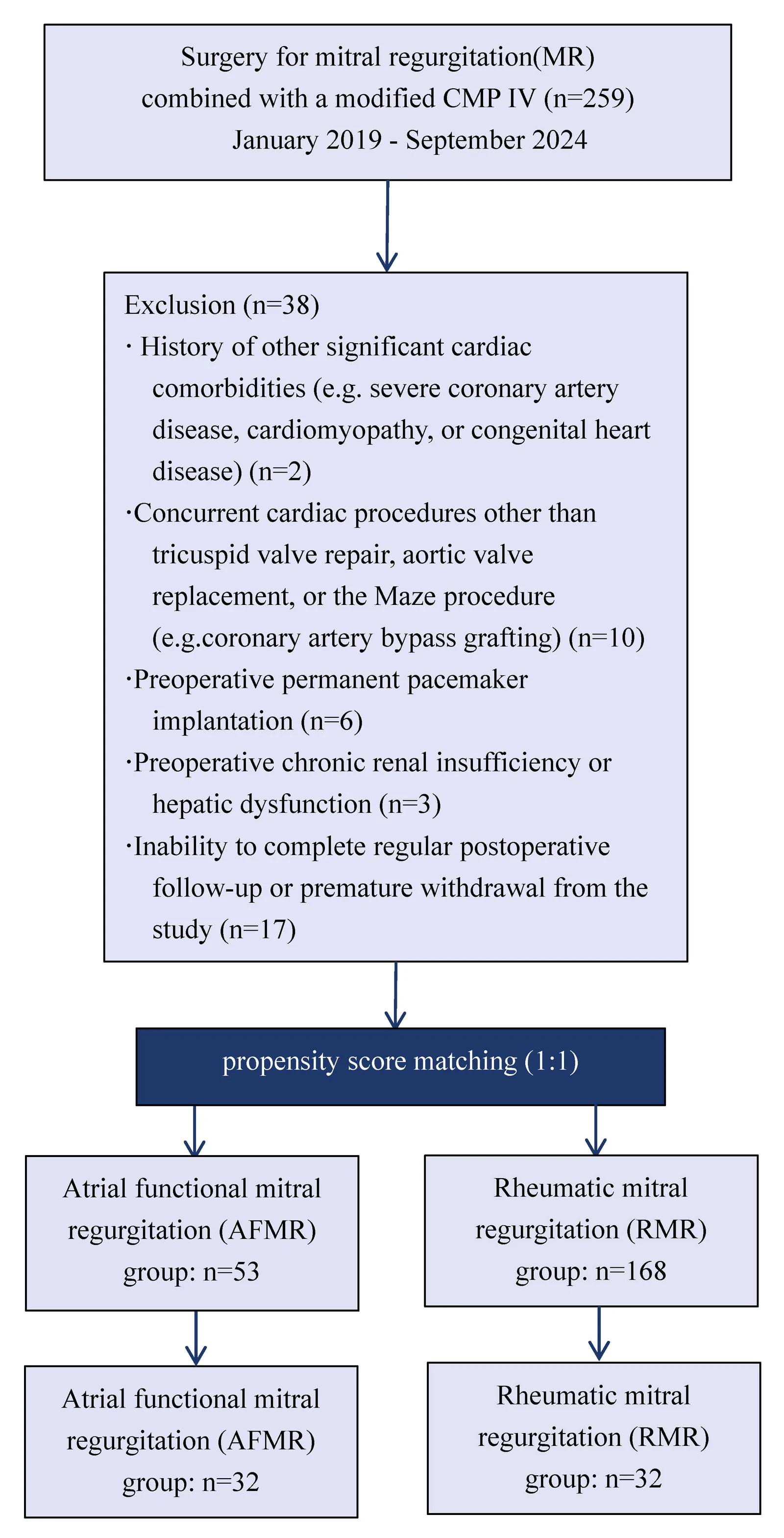
**Flowchart of patient selection and matching**. Abbreviations: MR, mitral regurgitation; CMP IV, Cox-Maze IV procedure; AFMR, atrial functional mitral regurgitation; RMR, rheumatic mitral regurgitation. A total of 259 patients underwent mitral valve surgery combined with the modified Cox-Maze IV procedure for MR and concomitant atrial fibrillation. The remaining 221 eligible patients were classified based on the etiology of mitral regurgitation: 53 patients in the AFMR group and 168 patients in the RMR group. To minimize selection bias and balance baseline covariates, 1:1 propensity score matching was performed, resulting in 32 well-matched pairs (32 patients in each group) for final analysis.

### 3.1 Patient Characteristics

Significant differences in age distribution were observed between the two groups (*p* < 0.001). Patients with RMR were significantly younger than those with AFMR (*p* < 0.001). The RMR group was predominantly female (79.8%). In contrast, the AFMR group showed a relatively balanced sex distribution (43.4% female). Body surface area (BSA) also differed significantly between groups (*p* < 0.001). The median BSA in the RMR group (n = 168) was 1.576 m^2^ (IQR: 0.19), which was significantly lower than the median of 1.695 m^2^ (IQR: 0.28) in the AFMR group (n = 53). Patients with rheumatic heart disease had a lower prevalence of hypertension. In contrast, patients in the AFMR group not only exhibited a higher prevalence of hypertension but also presented with significantly greater severity, as evidenced by a significantly higher proportion of patients with Grade Ⅲ hypertension (systolic blood pressure ≥180 mmHg) compared to the RMR group (AFMR: 18.9% vs. rheumatic mitral regurgitation (RMR): 4.8%). This finding suggests a potential association between AFMR and more severe hypertensive disease. Renal dysfunction was observed in 27.4% of RMR patients but was absent (0%) in the AFMR group. Compared with RMR, AFMR appeared to exhibit a “protective” effect on renal function in this cohort (*p* < 0.001) (Table 1).

Preoperative transthoracic echocardiography demonstrated significantly larger left ventricular end‑diastolic dimension (54.00 vs. 49.00 mm, *p* < 0.001), left atrial dimension (49.00 vs. 52.00 mm, *p* = 0.031), interventricular septal thickness (10.00 vs. 9.00 mm, *p* < 0.001), left ventricular posterior wall thickness (9.50 vs. 8.00 mm, *p* = 0.001), and mitral regurgitation area in the AFMR group compared with the RMR group (χ^2^ (4, N = 221) = 24.59, *p* < 0.001). Additionally, the aortic annulus size was significantly lager in the AFMR group than in the RMR group (33.22 ± 6.36 mm vs. 25.85 ± 6.20 mm, *p* = 0.007) (Table 2).

**Table 2.** **Preoperative echocardiographic characteristics**.

Preoperative TTE		RMR group (N = 168)	AFMR group (N = 53)	*p*-value
Mitral valve flow velocity, median (IQR) (m/s)		3.90 (2.65)	1.90 (3.55)	0.230
Mitral valve gradient, median (IQR), mmHg		64.0 (75)	14.50 (100)	0.222
Main pulmonary artery width, median (IQR), mm		23.00 (4)	23.00 (4)	0.655
Left atrial dimension, median (IQR), mm		52.00 (11)	49.00 (12)	0.031*
Left ventricular end-diastolic dimension, median (IQR), mm		49.00 (9)	54.00 (9)	0.001*
Interventricular septum thickness, median (IQR), mm		9.00 (2)	10.00 (2)	<0.001*
Left ventricular posterior wall thickness, median (IQR), mm		8.00 (1)	9.50 (2)	0.001*
Left ventricular ejection fraction, median (IQR), %		60.00 (9)	62.00 (7)	0.295
Mitral annulus size, mm		39.12 ± 7.045	42.67 ± 9.736	0.111
Aortic annulus size, mm		25.85 ± 6.20	33.22 ± 6.36	0.007*
Mitral regurgitation grade, n, %				<0.001*
	No regurgitation	25 (14.8)	0 (0)	
	Mild regurgitation	44 (26.1)	5 (9.4)	
	Moderate regurgitation	42 (25.0)	12 (22.6)	
	Moderate-severe regurgitation	22 (13.0)	14 (26.4)	
	Severe regurgitation	35 (20.8)	22 (41.5)	

Abbreviations: TTE, transthoracic echocardiography; RMR, rheumatic mitral regurgitation; AFMR, atrial functional mitral regurgitation; IQR, interquartile range. Notes: Data are presented as median (IQR), mean ± SD, or n (%). *p*-values were calculated using the Mann-Whitney U test for continuous variables with non-normal distribution, the independent samples *t*-test for normally distributed continuous variables, and the Chi-square test for categorical variables. An asterisk (*) indicates a statistically significant difference (*p* < 0.05). SD, standard deviation.

The incidence of acute kidney injury was significantly higher in the AFMR group compared to the RMR group (11.3% vs. 3.0%, *p* = 0.024). Furthermore, rates of both pre-discharge AF recurrence (32.0% vs. 11.3%, *p* < 0.001) and early AF recurrence (26.4% vs. 8.9%, *p* < 0.001) were markedly elevated in the AFMR group. No statistically significant differences were observed between the two groups regarding the rates of re-sternotomy, low cardiac output, permanent pacemaker implantation, 30-day mortality, cardiac mortality, or 1-year mortality (all *p* > 0.05) (Table 3).

**Table 3.** **Postoperative complications**.

Postoperative indicators	RMR group (N = 168)	AFMR group (N = 53)	*p*-value
Re-sternotomy, n (%)	4 (2.4)	2 (3.8)	0.629
Low cardiac output, n (%)	1 (0.6)	1 (1.9)	0.419
Acute kidney injury, n (%)	5 (3.0)	6 (11.3)	0.024*
Permanent pacemaker implantation, n (%)	1 (0.6)	0 (0)	1.000
30-day mortality, n (%)	3 (1.8)	2 (3.8)	0.596
Cardiac mortality, n (%)	4 (2.4)	2 (3.8)	0.632
1-year mortality, n (%)	5 (3.0)	2 (3.8)	0.674
Pre‑discharge AF recurrence, n (%)	19 (11.3)	17 (32.0)	<0.001*
Early atrial fibrillation recurrence, n (%)	15 (8.9)	14 (26.4)	<0.001*

Abbreviations: RMR, rheumatic mitral regurgitation; AFMR, atrial functional mitral regurgitation. Notes: Data are presented as n (%). *p*-values were calculated using the Chi-square test or Fisher’s exact test, as appropriate. An asterisk (*) indicates a statistically significant difference (*p* < 0.05).

After 1:1 propensity score matching based on statistically significant covariates identified in the univariate analysis (Tables 1,2,3), a total of 64 patients were included in the study, with 32 patients in the RMR group and 32 patients in the AFMR group (Table 4). Regarding demographic characteristics, sex is identical between groups, but the median ages are 60 and 63 years, no substantial residual imbalance was observed according to the selected balance criterion, after that criterion is clarified, and body surface area also showed no significant difference. In terms of comorbidities, the distribution of hypertension grades was well-balanced between groups (*p* = 0.806, SMD = 0.062). No patients in either group had renal dysfunction (SMD <0.001). All preoperative echocardiographic parameters showed no statistically significant differences between groups. These findings indicate that the baseline characteristics were balanced between the two groups after matching (Fig. 2).

**Table 4.** **Patient characteristics after 1:1 propensity score matching**.

Variable		RMR group (N = 32)	AFMR group (N = 32)	*p*-value	SMD
Age, median (IQR), years		60.00 (9)	63.00 (11)	0.761	0.076
Sex, female, n (%)		19 (59.4)	19 (59.4)	1.000	<0.001
BSA, median (IQR), m^2^		1.65 (0.19)	1.61 (0.26)	0.576	0.141
Hypertension, n (%)				0.806	0.062
	Grade Ⅰ	0 (0.0)	1 (3.1)		
	Grade Ⅱ	0 (0.0)	2 (6.3)		
	Grade Ⅲ	4 (12.5)	3 (9.4)		
Renal dysfunction, n (%)		0 (0.0)	0 (0.0)	-	<0.001
Left atrial diameter, median (IQR), mm		50.00 (8)	49.50 (15)	0.683	0.102
Interventricular septal thickness, median (IQR), mm		9.00 (2)	9.00 (3)	0.550	0.150
Left ventricular end-diastolic diameter, median (IQR), mm		54.00 (13)	53.00 (9)	0.965	0.011
Left ventricular posterior wall thickness, median (IQR), mm		9.00 (2)	9.00 (2)	0.924	0.024
Mitral regurgitation area, n (%)				0.749	0.080
	No regurgitation	1 (3.1)	0 (0)		
	Mild regurgitation	5 (15.6)	4 (12.5)		
	Moderate regurgitation	11 (34.3)	9 (28.1)		
	Moderate-severe regurgitation	1 (3.1)	9 (28.1)		
	Severe regurgitation	14 (43.7)	10 (31.2)		

Abbreviations: RMR, rheumatic mitral regurgitation; AFMR, atrial functional mitral regurgitation; IQR, interquartile range; BSA, body surface area; SMD, standardized mean difference. Notes: Data are presented as median (IQR) or n (%). A *p*-value < 0.05 was considered statistically significant. *p*-value was not calculated for renal dysfunction due to no events in either group. Standardized mean differences (SMDs) were calculated to assess balance between groups, SMD <0.2 as the prespecified criterion for acceptable balance.

**Fig. 2.**
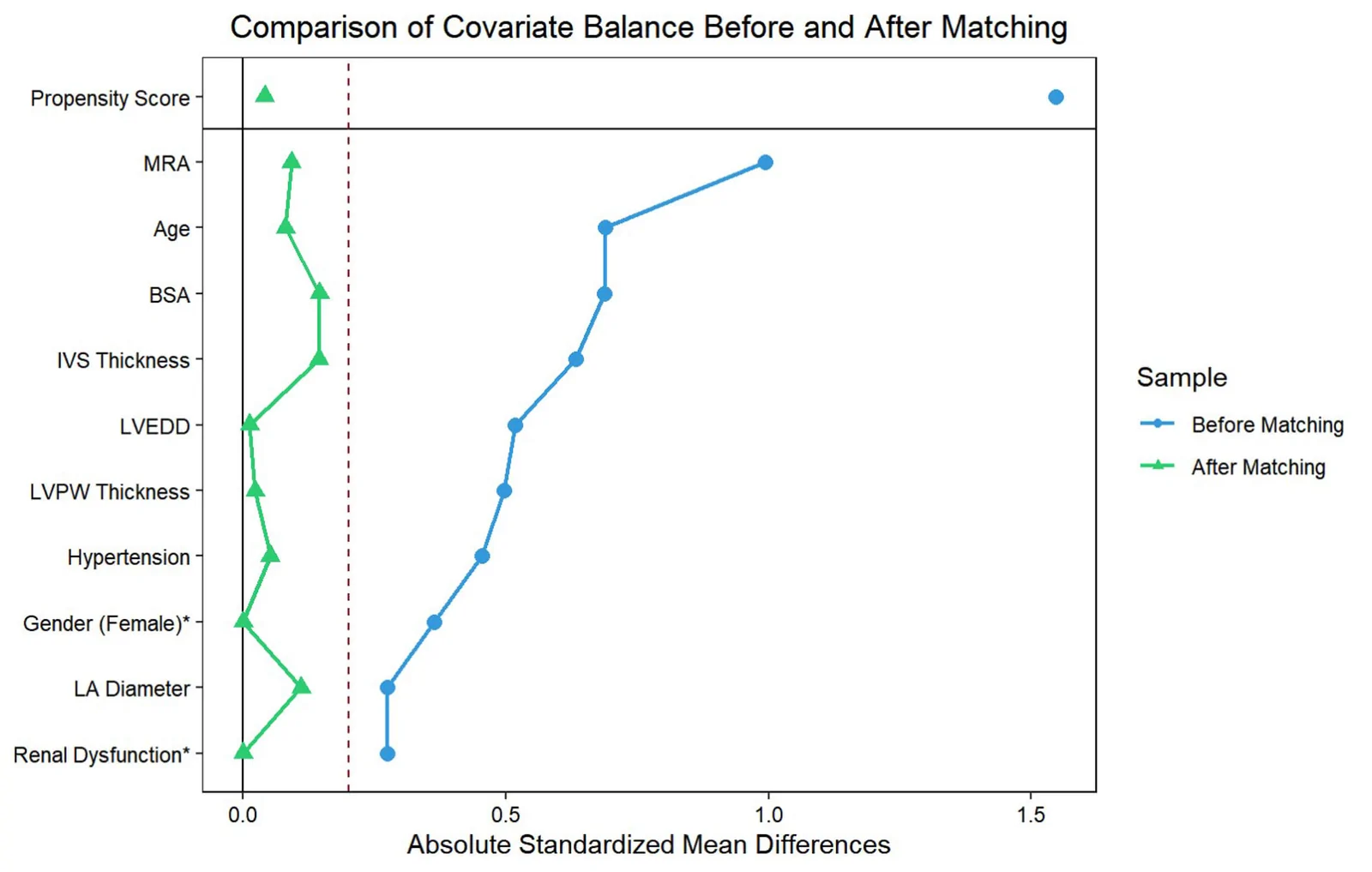
**Covariate balance plot before and after propensity score matching**. Abbreviations: SMD, standardized mean difference; MRA, Mitral regurgitation area; BSA, body surface area; IVS, interventricular septum; LVEDD, left ventricular end-diastolic diameter; LVPW, left ventricular posterior wall; LA, left atrial. This figure displays the absolute standardized mean differences (SMD) for each covariate before and after 1:1 propensity score matching. Blue circles represent the unmatched cohort, and green triangles represent the matched cohort. The X-axis indicates the absolute SMD, with a dashed line typically drawn at 0.2 to denote acceptable balance. After matching, all covariates, including propensity score, demonstrated substantially reduced SMDs compared to the pre-match sample, indicating that the matching procedure successfully balanced the distribution of observed baseline characteristics between the two groups. Covariates marked with an asterisk (*) include gender and renal dysfunction, which showed notable improvement in balance after matching.

### 3.2 Primary Endpoint (Matched Cohort Comparisons)

#### 3.2.1 Postoperative AF recurrence rate

Overall, the risk of postoperative atrial fibrillation recurrence was higher in the AFMR group than in the RMR group. The AF recurrence rate before discharge was 37.5% in the AFMR group versus 12.5% in the RMR group (*p* = 0.057). At 1‑year follow‑up, the recurrence rate remained significantly higher in the AFMR group (31.2% vs. 6.2%, *p* = 0.021) (Table 5).

**Table 5.** **Intraoperative and postoperative indicators after propensity score matching**.

Intraoperative & postoperative indicators	RMR group (N = 32)	AFMR group (N = 32)	*p*-value
Concomitant aortic valve surgery, n, %	19 (59.4)	15 (46.9)	0.316
Concomitant tricuspid valve surgery, n, %	27 (84.4)	26 (81.3)	0.740
Mitral valve replacement, n, %	31 (96.9)	27 (84.4)	0.196
Cardiopulmonary bypass time, median (IQR), min	188.5 (45.5)	191 (46.5)	0.478
Aortic cross-clamp time, median (IQR), min	125 (35.5)	125 (40.0)	0.649
Assisted circulation time, median (IQR), min	40 (8.5)	43 (10.0)	0.127
Re-sternotomy, n, %	1 (3.1)	0 (0)	1.000
Acute kidney injury, n, %	1 (3.1)	3 (9.4)	0.613
Pre‑discharge AF recurrence, n (%)	4 (12.5)	12 (37.5)	0.057
Early AF recurrence, n (%)	2 (6.2)	10 (31.2)	0.021
Permanent pacemaker implantation, n, %	0 (0)	0 (0)	-
Aortic annulus size, mm	24.56 ± 5.59	31.75 ± 6.07	0.365
30-day mortality, n, %	0 (0)	1 (3.1)	1.000
1‑year mortality, n, %	1 (3.1)	1 (3.1)	1.000

Abbreviations: RMR, rheumatic mitral regurgitation; AFMR, atrial functional mitral regurgitation; IQR, interquartile range. Notes: Data are presented as mean ± SD for normally distributed variables and as median (IQR) for non‑normally distributed variables. Normality was assessed using the Shapiro–Wilk test. *p*-values for the available paired samples using the McNemar, Fisher, paired *t*-tests, and Wilcoxon signed-rank tests.

#### 3.2.2 Analysis of Intraoperative Variables

In the AFMR group, 46.88% of patients underwent concurrent aortic valve surgery, compared with 59.38% in the RMR group (*p* = 0.316). Tricuspid valve surgery was performed in 81.25% of AFMR patients and 84.38% of RMR patients (*p* = 0.740). No statistically significant difference was observed between the two groups with respect to the type of mitral valve procedure (repair vs. replacement; *p* = 0.196).

#### 3.2.3 Timing of Postoperative AF recurrence and survival analysis

Kaplan–Meier analysis showed a significant difference in AF‑free survival between the groups (log‑rank χ^2^ = 6.33, *p* = 0.012). The RMR group demonstrated a significantly higher rate of freedom from recurrence compared to the AFMR group (Fig. 3). Postoperatively, one death occurred in each group after matching, with no statistically significant difference between the groups (*p* = 1.000).The log-rank test showed no significant difference in survival between the two groups (*p* = 0.99). The median follow-up time was 26 months (IQR: 13–31.75 months; range: 5–74 months) in the RMR group and 25 months (IQR: 14.25–49.25 months; range: 0–77 months) in the AFMR group (Fig. 4).

**Fig. 3.**
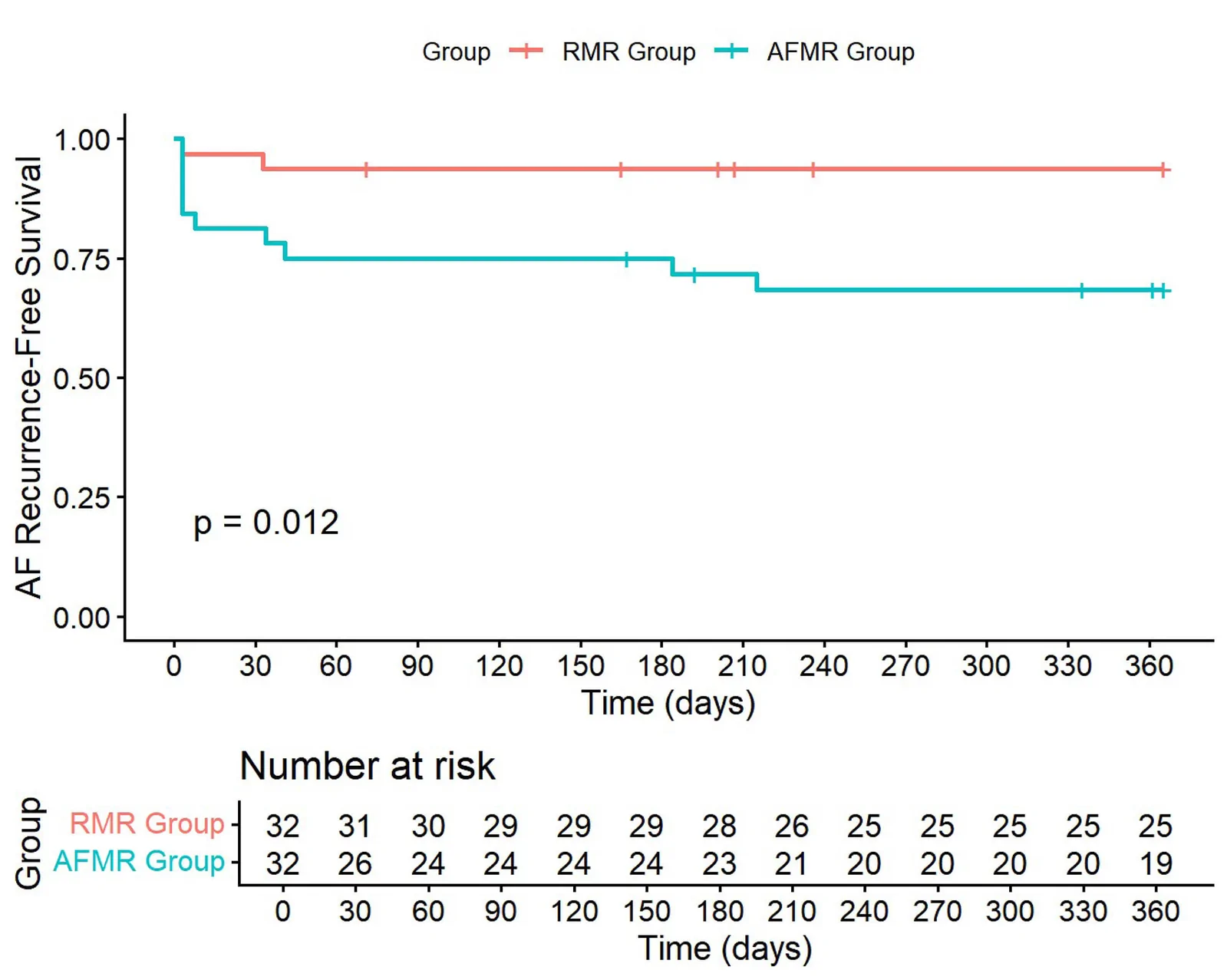
**Kaplan-Meier curves for freedom from atrial fibrillation (AF) recurrence**. The curves depict the probability of remaining free from atrial fibrillation (AF) recurrence over the 12-month postoperative period for patients with rheumatic mitral regurgitation (RMR, red line) and atrial functional mitral regurgitation (AFMR, Green line). Recurrence was defined as any AF, documented by two consecutive ECGs or 24‑hour Holter monitors between discharge and 12 months post‑operatively. Patients without the event were censored at the last follow‑up, death, or study cutoff (September 2025), whichever occurred first. The number of patients at risk at each time point is shown below the graph.

**Fig. 4.**
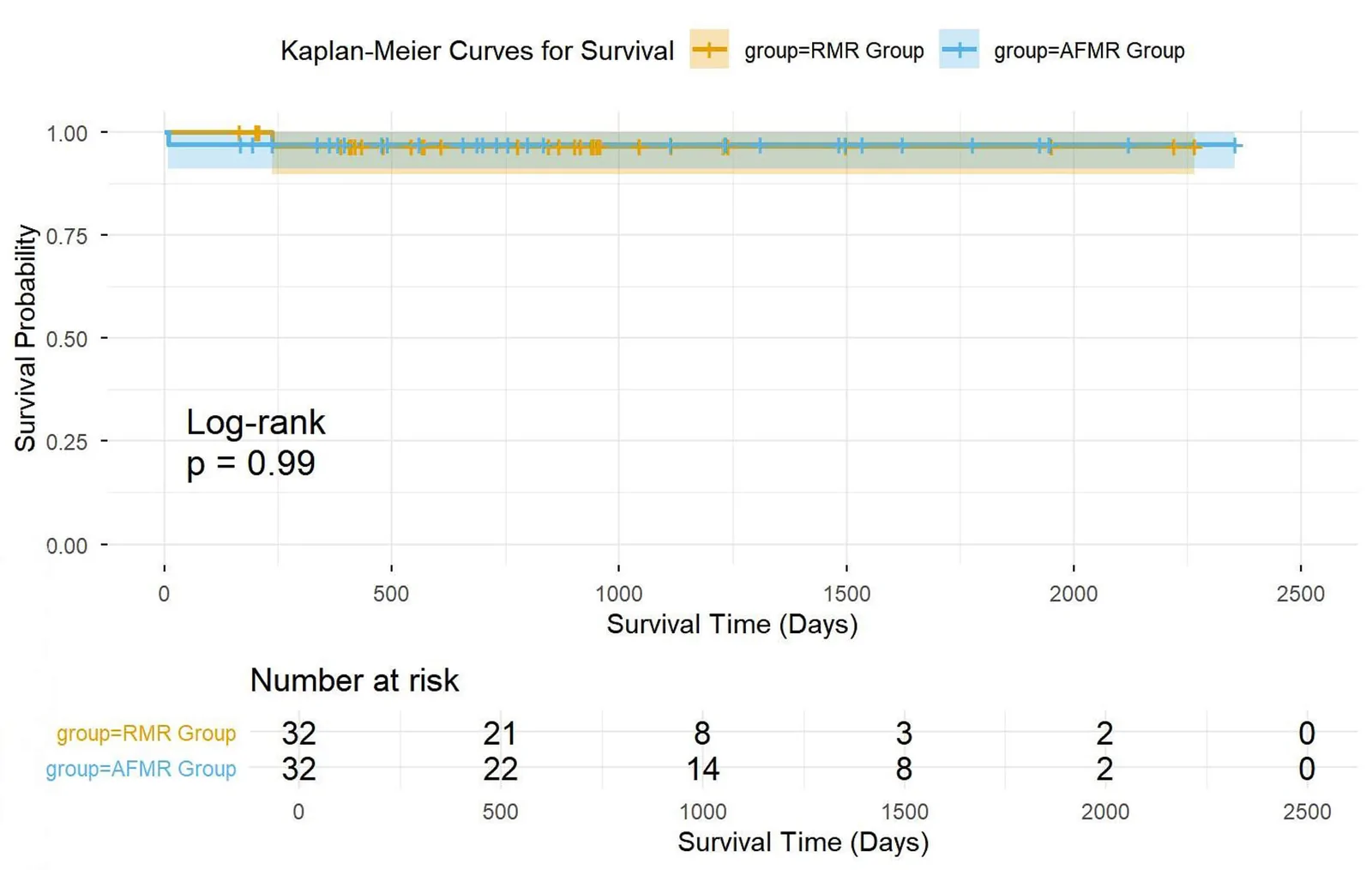
**Kaplan-Meier curves for overall survival comparing RMR and AFMR groups**. Kaplan-Meier survival analysis was performed to compare overall survival between the RMR group (yellow line) and the AFMR group (blue line). The number of patients at risk at each time point is shown below the figure. Censored events are indicated by tick marks on the curves. Observed overall survival did not differ significantly between the AFMR and RMR groups. The survival curve extends to the maximum observed follow‑up of approximately 2500 days.

The results of the sensitivity analysis were largely consistent with those of the primary analysis (Table 6). After excluding patients who experienced recurrence during the blanking period, 5 patients (18.5%) in the AFMR group experienced atrial fibrillation recurrence, whereas there were no recurrences in the RMR group (0%); the difference in survival curves between the two groups was statistically significant (χ^2^ = 6.01, *p* = 0.014). An analysis treating recurrences during the blanking period as missing data yielded consistent results (χ^2^ = 5.63, *p* = 0.018). A left-censored analysis starting 3 months postoperatively showed that 2 patients (6.2%) in the AFMR group relapsed after 3 months, while there were no relapses in the RMR group; the difference was close to statistical significance (χ^2^ = 3.29, *p* = 0.070), which may be related to the small sample size. All sensitivity analyses indicated that the risk of AF recurrence was higher in the AFMR group than in the RMR group, suggesting that the results of this study are robust.

**Table 6.** **Sensitivity analysis of atrial fibrillation recurrence (including a 3-month blank period)**.

Analysis method	RMR group (N = 32)	AFMR group (N = 32)	*Statistic*	*p*-value
Primary analysis, %	2/32 (6.2%)	10/32 (31.2%)	OR = 6.63 (1.23–68.27)	0.021
Excluding patients with recurrence during the blank period^1^, %	0/30 (0%)	5/27 (18.5%)	χ^2^ = 6.01	0.014
Using 3 months as the starting point^2^, %	0/32 (0%)	2/32 (6.2%)	χ^2^ = 3.29	0.070
Recurrence during the blank period treated as missing^3^, %	0/32 (0%)	5/32 (15.6%)	χ^2^ = 5.63	0.018

Notes:^1^ Excludes patients who relapsed within 3 months post-surgery; ^2^ Uses 3 months post-surgery as the start of follow-up, a left‑censored landmark analysis with a different risk set definition (only patients event‑free at exactly 3 months are included), and therefore addresses a distinct analytical question; ^3^ Patients with recurrence during the blank period are treated as missing, the event counts are not directly comparable across analyses due to differences in risk set definitions and censoring rules.

### 3.3 Secondary Endpoints

Postoperative Permanent Pacemaker Implantation: During the 1‑year postoperative period, one patient in the RMR group required permanent pacemaker implantation before matching, whereas no patient in either group required permanent pacemaker implantation after matching. Both groups exhibited a similar degree of cardiac reverse remodeling. The left ventricular end‑diastolic diameter decreased in both groups, but the change was not statistically significant (*p* = 0.861). Similarly, no significant changes were observed in interventricular septal thickness (*p* = 0.103), left atrial diameter (*p* = 0.441), or left ventricular posterior wall thickness (*p* = 0.752) (Table 7).

**Table 7.** **Preoperative echocardiographic parameters and pre- and postoperative changes (delta values) in the propensity score-matched cohort**.

Echocardiographic parameter	RMR Group (N = 32)	AFMR Group (N = 32)	*p*-value
Main pulmonary artery width, median (IQR), mm	24.00 (4)	24.00 (5)	0.032
Left atrial dimension, mean ± SD, mm	50.00 (8)	49.50 (15)	0.683
Interventricular septum thickness, median (IQR), mm	9.00 (2)	9.00 (3)	0.550
Left ventricular end-diastolic dimension, median (IQR), mm	54.00 (13)	53.00 (9)	0.965
Left ventricular posterior wall thickness, median (IQR), mm	9.00 (2)	9.00 (2)	0.924
Left ventricular ejection fraction, median (IQR), %	57.50 (15)	59.00 (8)	0.622
Delta main pulmonary artery width, median (IQR), mm	0.00 (6)	–0.50 (6)	0.967
Delta left atrial dimension, median (IQR), mm	–8.00 (12)	–8.50 (9)	0.441
Delta interventricular septum thickness, median (IQR), mm	1.00 (2)	0.00 (2)	0.103
Delta left ventricular end-diastolic dimension, median (IQR), mm	–5.50 (8)	–5.00 (8)	0.861
Delta left ventricular posterior wall thickness, mean ± SD, mm	0.42 ± 1.29	0.39 ± 1.28	0.752
Delta left ventricular ejection fraction, median (IQR), %	–1.00 (10)	–1.50 (13)	0.736

Abbreviations: AFMR, atrial functional mitral regurgitation; RMR, rheumatic mitral regurgitation; IQR, interquartile range; Delta, postoperative change. Notes: With the exception of a slight statistical difference in main pulmonary artery width, preoperative parameters were generally comparable between the two groups. There were no significant differences in the between-group comparisons of all Delta parameters (all *p* > 0.05), indicating that the degree of postoperative cardiac remodeling was similar between the two groups.

### 3.4 Risk Factor Analysis

Compared with RMR patients, those in the AFMR group had a significantly higher risk of AF recurrence, and this risk increased progressively over time — from 3.67-fold before discharge to 9.00-fold at 1 year postoperatively. Conditional logistic regression, which was selected as the primary analytical approach for the matched cohort, demonstrated that the AFMR group was associated with a higher risk of AF recurrence both before discharge (OR = 3.67, 95% CI: 1.02–13.14; *p* = 0.046) and at 1‑year follow‑up (OR = 9.00, 95% CI: 1.14–71.04; *p* = 0.037). The widening ORs and confidence intervals over time reflect the cumulative nature of the recurrence risk rather than statistical instability, as the number of events in the RMR group remained very low during follow‑up, which inherently leads to wider CIs for the treatment effect in matched analyses. Cox proportional hazards models yielded consistent findings, further supporting the robustness of the primary conditional logistic regression results (**Supplementary Table 1**).

Left atrial diameter emerged as an independent risk factor for AF recurrence (hazard ratios (HR) = 1.05, 95% CI: 1.01–1.10, *p* = 0.021). Age was also independently associated with recurrence (HR = 1.06, 95% CI: 1.00–1.12, *p* = 0.038). Group assignment showed a trend toward increased risk but did not reach statistical significance (HR = 2.68, 95% CI: 0.86–8.39, *p* = 0.089). Mitral annulus diameter was not significantly associated with recurrence (HR = 1.03, 95% CI: 0.95–1.11, *p* = 0.518) (Fig. 5).

**Fig. 5.**
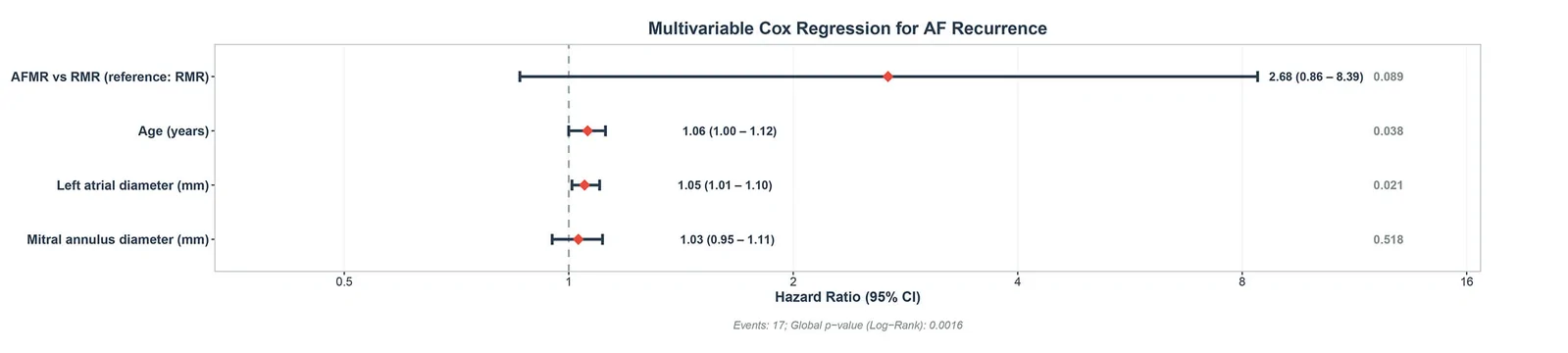
**Forest plot of multivariable Cox regression analysis for predictors of atrial fibrillation recurrence after ablation**. Multivariable Cox regression analysis was performed to identify independent predictors of AF recurrence, including all 221 patients (17 events). Hazard ratios (HR) and 95% confidence intervals (CI) are shown for each variable. The vertical dashed line indicates an HR of 1.0 (no effect). Variables with HR >1 indicate an increased risk of recurrence. The model demonstrated good discriminative ability (C-index = 0.744) and satisfied the proportional hazards assumption (global *p* = 0.254). The global log-rank test was significant (*p* = 0.0016), indicating that the model effectively predicted AF recurrence.

### 3.5 Surgical Outcomes in Patients With AFMR

Preoperative and postoperative TTE was used to assess cardiac remodeling. Postoperative measurements showed a significant reduction in Left ventricular end-diastolic dimension (54.00 vs. 48.00; *p* < 0.001) and a significant change in left atrial dimension (*p* < 0.001). In contrast, no significant differences were observed between preoperative and postoperative values in main pulmonary artery diameter (*p* = 0.474), interventricular septal thickness (*p* = 0.469), left ventricular posterior wall thickness (*p* = 0.174), or left ventricular ejection fraction (*p* = 0.569) (Table 8).

**Table 8.** **Changes in echocardiographic parameters before and after surgery in the AFMR group**.

Echocardiographic parameter	Preoperative	Postoperative	Difference	*p*-value
Main pulmonary artery width, median (IQR), mm	23.00 (4)	23.00 (3)	–0.68	0.474
Left atrial dimension, median (IQR), mm	49.00 (12)	45.00 (6)	–7.71	<0.001
Interventricular septum thickness, median (IQR), mm	10.00 (2)	10.00 (1)	–0.22	0.469
Left ventricular end-diastolic dimension, median (IQR), mm	54.00 (9)	48.00 (8)	–3.96	<0.001
Left ventricular posterior wall thickness, median (IQR), mm	9.50 (2)	10.00 (1)	0.24	0.174
Left ventricular ejection fraction, median (IQR), %	62.00 (7)	57.00 (7)	–0.49	0.569

Abbreviations: IQR, interquartile range. Notes: Data are presented as median (interquartile range, IQR). The difference column represents the change from preoperative to postoperative values (postoperative−preoperative).

## 4. Discussion

### 4.1 Pathological Differences Between AFMR and RMR

First, regarding differences in the risk of recurrence specific to the underlying cause. The results of this study show that the risk of atrial fibrillation recurrence after modified Cox-Maze IV surgery is significantly higher in patients with AFMR than in those with RMR. This finding may be closely related to the distinct underlying pathophysiological mechanisms of the two conditions. RMR primarily stems from structural damage to the valve itself caused by rheumatic heart disease, whereas the core of AFMR lies in the functional remodeling of the left atrium and left ventricle [12]. In patients with long‑standing persistent AF, the progression of atrial cardiomyopathy may be particularly severe and less reversible [13]. Furthermore, AF recurrence may also be closely related to functional damage to the mitral valve. Prolonged duration of AF leads to more complex interactions between the atria and the mitral valve, resulting in a vicious cycle of mitral valve insufficiency and atrial dilation. In contrast, patients with rheumatic mitral regurgitation, despite having more severe structural damage to the mitral valve, exhibit a relatively stable atrial electrophysiological environment. Existing studies have shown that the Cox‑Maze IV procedure exhibits comparable efficacy in restoring sinus rhythm within five years among patients with rheumatic and degenerative mitral valve disease [14]. In contrast, other studies indicate that patients with AFMR and concomitant AF may face a higher risk of requiring permanent pacemaker implantation following maze surgery [13]. Collectively, these findings imply that for AFMR patients, mitral valve surgery alone may be insufficient to eliminate the risk of AF recurrence. Therefore, peri‑ or postoperative strategies specifically targeting the atrial substrate—such as more extensive ablation sets or optimized antiarrhythmic therapy—hold significant clinical potential for improving long‑term rhythm outcomes.

In patients with AFMR, there is a common cyclical relationship in which “atrial fibrillation—atrial enlargement—mitral regurgitation—further atrial fibrillation” reinforce one another. Long-term atrial fibrillation leads to a loss of effective atrial contractility, resulting in left atrial enlargement and an enlarged annulus, which exacerbates regurgitation; the mitral annulus gradually dilates and flattens, reducing the area of valve leaflet overlap and thereby causing functional regurgitation. Mitral regurgitation further increases the volume load on the left atrium, accelerating atrial structural remodeling. Increased atrial wall tension can activate multiple signaling pathways, including the TGF-β pathway and the release of inflammatory factors, promoting myocardial fibrosis and extracellular matrix deposition. This positive feedback mechanism makes it difficult for patients with AFMR to maintain sinus rhythm long-term, even if they regain it in the short term after surgery. Additionally, AF itself can reduce the effective atrial refractory period and increase electrical conduction heterogeneity through an “electrical remodeling” mechanism, making the atria more prone to sustaining re-entrant activity—a phenomenon known as “AF begets AF”.

In this process, patients with AFMR are often in the “late stage” of the disease, with relatively severe structural and functional damage to the atria. Therefore, even after surgical restoration of valve function, atrial remodeling is difficult to fully reverse, leading to a high postoperative recurrence rate.

In this study, both groups of patients showed a trend toward improvement in postoperative cardiac structure (such as left atrial and left ventricular diameters); however, the AFMR group still exhibited a higher recurrence rate, further supporting the notion that “structural reversal ≠ electrophysiological reversal”.

### 4.2 Efficacy and Limitations of the Modified Cox-Maze IV Procedure

As the gold standard procedure for treating AF, the Cox-Maze IV procedure has demonstrated good efficacy in numerous studies. The modified Cox-Maze IV procedure currently used at our institution, which incorporates the “cut-and-sew” technique, has been shown to yield better outcomes than the traditional procedure. However, this study indicates that while the Cox-Maze IV procedure effectively alleviates symptoms of atrial fibrillation and improves the rate of postoperative restoration of sinus rhythm, its efficacy is inferior in patients with AFMR compared to those with RMR. This may be related to the complex electrophysiological environment and functional mitral valve damage in AFMR patients.

In AFMR patients, although atrial ablation during surgery can eliminate abnormal electrical activity within the atria, the long-term persistence of atrial fibrillation has already led to changes in atrial electrophysiology, making it difficult to restore sinus rhythm postoperatively. Furthermore, these electrophysiological alterations in the atria result in the Cox-Maze IV procedure yielding less-than-expected outcomes, presenting a challenge for future treatment strategies.

Therefore, for patients with AFMR, the Cox-Maze IV procedure may need to be combined with other therapeutic approaches—such as expanded ablation areas, more advanced rhythm-control medications, or novel atrial electrophysiological therapies-to improve postoperative atrial fibrillation control rates.

### 4.3 Predictors of Atrial Fibrillation Recurrence

Multivariable analysis confirmed that preoperative left atrial enlargement and age are independent risk factors for postoperative AF recurrence, a finding consistent with existing literature. Left atrial size is a well‑established imaging marker of atrial remodeling and AF burden [15,16,17,18,19]. Our results reinforce its value in preoperative risk stratification. Beyond indicating disease severity, left atrial dimensions may also inform the selection of ablation strategy. For instance, whether more extensive posterior left atrial isolation is beneficial in this population merits further study. In patients with severe left atrial enlargement due to valvular disease and AF, we have attempted combined modified Cox Maze IV procedure with left atrial volume reduction; however, postoperative sinus rhythm maintenance rates remained suboptimal. Histological examination of resected atrial tissue from these cases revealed extensive myocardial fibrosis. Future studies could explore whether more aggressive adjunct ablation of fibrotic areas in patients with valvular disease and AF who have significantly enlarged left atria could further improve the rate of sinus rhythm maintenance.

### 4.4 Surgical Safety and Survival Benefits

In the present study, 30‑day, early, and mid‑term mortality rates were low in both groups, with no significant intergroup differences. Rates of permanent pacemaker implantation were also low in both cohorts and did not show a clinically meaningful increase over time. Collectively, these findings lend strong support to the safety and efficacy of a modified Cox‑Maze IV procedure as a concomitant surgical intervention in eligible patients with mitral regurgitation and atrial fibrillation, irrespective of the underlying etiology [20,21,22]. This evidence reinforces the rationale for broader adoption of this procedure in routine clinical practice for patients with mitral regurgitation and coexistent atrial fibrillation, particularly among those with recently defined atrial functional mitral regurgitation. Nevertheless, it should be acknowledged that despite the favorable rhythm outcomes achieved by surgery, restoration of sinus rhythm does not necessarily translate into proportional improvements in overall prognosis. Further investigation is warranted to comprehensively evaluate post‑operative stroke risk, changes in cardiac function, and long‑term survival benefits in this patient population.

### 4.5 Study Limitations

This study is a single-center, retrospective cohort study with a small sample size and a single data source, which may introduce selection bias. Although propensity score matching was used to balance baseline differences as much as possible, the influence of confounding factors could not be completely eliminated. Therefore, the study results may not fully represent the situation in other regions, particularly among patients with different demographic backgrounds. Furthermore, the small sample size after matching and the limited number of events resulted in wide confidence intervals for some risk estimates, which somewhat compromised the stability of the results. Additionally, due to sample size constraints, detailed subgroup analyses could not be conducted. This study did not include more refined structural indicators, such as the degree of atrial fibrosis, nor did it perform a systematic analysis of postoperative pharmacotherapy (oral anticoagulants and antiarrhythmic drugs). Future multicenter, large-sample prospective studies will offer greater representativeness and broader applicability.

Furthermore, monitoring of postoperative AF primarily relied on routine electrocardiograms, which may have underestimated the recurrence of asymptomatic AF. Additionally, although we followed up on AF recurrence for 1 year postoperatively, long-term follow-up data are lacking. The relatively limited follow-up period precludes an evaluation of long-term rhythm outcomes. With a median follow-up duration of only 2 years, further research is needed to assess the long-term implications of AF recurrence, such as long-term survival rates, stroke risk, and valve durability. These factors are crucial for evaluating surgical efficacy and the effectiveness of postoperative management strategies.

Although this study found a statistically significant difference in aortic valve size between the two patient groups, which may have some impact on the risk of AF recurrence, we did not conduct an in-depth analysis of the specific effects of the aortic valve due to sample size limitations. Future studies should conduct a more detailed assessment of factors such as aortic annulus size and valve calcification to clarify their roles in patients with AFMR.

## 5. Conclusions

This study demonstrates that, although early postoperative survival was comparable between patients with AFMR and those with RMR following the Cox‑Maze IV procedure, the AFMR group showed a higher risk of AF recurrence over the follow‑up period. Notably, the between‑group difference in AF recurrence did not reach statistical significance at the pre‑discharge assessment, while it became more apparent during later follow‑up. These findings suggest that the elevated recurrence risk observed in AFMR patients is primarily associated with the clinical and echocardiographic characteristics inherent to this population. In particular, parameters such as enlarged left atrial diameter and advanced age emerged as important predictors of recurrence, highlighting the need for comprehensive preoperative risk stratification. For AFMR patients, a more tailored surgical approach—potentially incorporating extended ablation lesions to address the remodeled atrial substrate, along with optimized postoperative rhythm management—may be warranted. Future efforts should focus on refining patient selection and ablation strategies to improve long‑term rhythm outcomes in this high‑risk subgroup.

## Data Availability

De-identified participant data that underpin the results reported in this article will be made available to qualified researchers for non-commercial purposes, following review and approval of a methodologically sound proposal. Data requests should be directed to the corresponding author and will require a signed data access agreement.

## Abbreviations

AF, atrial fibrillation; AFMR, atrial functional mitral regurgitation; RMR, rheumatic mitral regurgitation; CMP IV, Cox-Maze IV procedure; VFMR, ventricular functional mitral regurgitation; MVD, mitral valve disease; DMR, degenerative mitral regurgitation; ECG, electrocardiograms; Scr, serum creatinine; TTE, transthoracic echocardiography; BSA, Body surface area; SMD, Standardized mean differences; LA, left atrial; PSM, propensity score matching.
