# Supplementary material for: Risk of Atrial Fibrillation Recurrence After Modified Cox-Maze IV Surgery: Comparing Atrial Functional Mitral Regurgitation and Rheumatic Mitral Regurgitation
Source: Rev Cardiovasc Med. 2026 Sep 7;27(9):49184. doi: 10.31083/RCM49184 (PMC13653180; doi:10.31083/RCM49184)
Supplement: Supplementary file 1 [file 2153-8174-27-9-49184-s1.zip › Supplementary Table 1.docx]

Supplementary Table 1. Comparison of AF Recurrence Risk Between AFMR and RMR Groups Using Different Statistical Methods.

| Outcome | Method | OR/HR | 95% CI | *p*-value |
| --- | --- | --- | --- | --- |
| Recurrence of atrial fibrillation before discharge | Matching-ignored logistic | 4.42 | 1.32–17.75 | 0.0221 |
| Recurrence of AF before discharge | Conditional logistic | 3.67 | 1.02–13.14 | 0.0461 |
| Recurrence of AF before discharge | Cox proportional hazards model (matched) | 3.00 | 0.97–9.3 | 0.0571 |
| Recurrence of AF during early follow-up | Matching-ignored logistic | 6.82 | 1.6–47.33 | 0.0198 |
| Recurrence of AF during early follow-up | Conditional logistic | 9.00 | 1.14–71.04 | 0.0371 |
| Recurrence of AF during early follow-up | Cox proportional hazards model (matched) | 5.00 | 1.1–22.82 | 0.0377 |

Notes: OR, odds ratio, reported for logistic regression models; HR, hazard ratio, reported for Cox proportional hazards model. Matching-ignored logistic regression did not account for the paired design from propensity score matching. Conditional logistic regression and Cox proportional hazards model (with paired stratification) both accounted for the paired design.
